# Carriere's "Hesitating Pulse" in Pneumonia

**Published:** 1909-04-17

**Authors:** 


### CARRIÈRE'S "HESITATING PULSE" IN PNEUMONIA.

The different characters that the pulse may present in ordinary lobar pneumonia, and the prognostic value of each type, have been much discussed. Very rightly have the frequency of the pulse, its rhythm, its volume and force, its dicrotism, and its tension all been insisted upon as important. Dr. Carrière lays particular stress upon one additional feature not often referred to in the books, and termed by him the "hesitating" character of the pulse. The epithet almost explains itself. In a case in which the pulse "hesitates" it seems as if the heart wavered at the time of ejecting its contents into the arterial system, and this wavering or hesitation can be readily felt by the finger and seen upon sphygmographic tracings. To the finger the radial artery, instead of becoming distended briskly after the commencement of the pulse-wave, becomes raised progressively and slowly, as though regretfully and with great effort. Precisely the same slowness in attaining the maximum systolic elevation is quite obvious in graphic records of the pulse. In a normal sphygmogram the ascending line is not quite vertical, but very nearly so. In cases of pulse "hesitancy," on the contrary, the ascending line is quite oblique. It is not a very common phenomenon fortunately, for it is of bad prognostic significance. Dr. Carrière met with it in six out of 28 cases of severe pneumonia. Of these six patients, five died. The phenomenon is best marked in the afternoon and evening from 3 to 6 P.M. It may occur by the fourth day of the disease; Dr. Carrière has not observed it before the fourth day, and it may not appear until the sixth day or later. It usually coincides with a minimum arterial tension. In the fatal cases there was always segmentary myocarditis.

